# MicroRNA181a Is Overexpressed in T-Cell Leukemia/Lymphoma and Related to Chemoresistance

**DOI:** 10.1155/2015/197241

**Published:** 2015-09-07

**Authors:** Zi-Xun Yan, Zhong Zheng, Wen Xue, Ming-Zhe Zhao, Xiao-Chun Fei, Li-Li Wu, Li-Min Huang, Christophe Leboeuf, Anne Janin, Li Wang, Wei-Li Zhao

**Affiliations:** ^1^State Key Laboratory of Medical Genomics, Shanghai Institute of Hematology, Shanghai Ruijin Hospital, School of Medicine, Shanghai Jiao Tong University, Shanghai 200025, China; ^2^French-Chinese Laboratory of Genomic and Life Sciences, Laboratory of Molecular Pathology, Shanghai 200025, China; ^3^Department of Hematology, Central Hospital of Jinhua, Zhejiang 321000, China; ^4^Department of Pathology, Shanghai Ruijin Hospital, School of Medicine, Shanghai Jiao Tong University, Shanghai 200025, China; ^5^Department of Oncology, People's Hospital of Guizhou Province, Guiyang 550001, China; ^6^Laboratory of Pathology, Paris Diderot University, U1165 Inserm, 75010 Paris, France

## Abstract

MicroRNAs (miRs) play an important role in tumorogenesis and chemoresistance in lymphoid malignancies. Comparing with reactive hyperplasia, miR181a was overexpressed in 130 patients with T-cell leukemia/lymphoma, including acute T-cell lymphoblastic leukemia (*n* = 32), T-cell lymphoblastic lymphoma (*n* = 16), peripheral T-cell lymphoma, not otherwise specified (*n* = 45), anaplastic large cell lymphoma (*n* = 15), and angioimmunoblastic T-cell lymphoma (*n* = 22). Irrespective to histological subtypes, miR181a overexpression was associated with increased AKT phosphorylation. *In vitro*, ectopic expression of miR181a in HEK-293T cells significantly enhanced cell proliferation, activated AKT, and conferred cell resistance to doxorubicin. Meanwhile, miR181a expression was upregulated in Jurkat cells, along with AKT activation, during exposure to chemotherapeutic agents regularly applied to T-cell leukemia/lymphoma treatment, such as doxorubicin, cyclophosphamide, cytarabine, and cisplatin. Isogenic doxorubicin-resistant Jurkat and H9 cells were subsequently developed, which also presented with miR181a overexpression and cross-resistance to cyclophosphamide and cisplatin. Meanwhile, specific inhibition of miR181a enhanced Jurkat and H9 cell sensitivity to chemotherapeutic agents, further indicating that miR181a was involved in acquired chemoresistance. Collectively, miR181a functioned as a biomarker of T-cell leukemia/lymphoma through modulation of AKT pathway. Related to tumor cell chemoresistance, miR181a could be a potential therapeutic target in treating T-cell malignancies.

## 1. Introduction

Malignancies derived from the T-cell lineages encompass a heterogeneous group of neoplasm. The World Health Organization (WHO) classification recognizes distinctive subtypes of immature T-cell malignancies, like acute T-cell lymphoblastic leukemia (T-ALL) and T-cell lymphoblastic lymphoma (T-LBL), as well as mature T-cell malignancies, mainly including peripheral T-cell lymphoma, not otherwise specified (PTCL-NOS), anaplastic large cell lymphoma (ALCL), and angioimmunoblastic T-cell lymphoma (AITL) [1]. Varied from clinicopathological features and biological behavior, they are generally more aggressive than their B-cell counterpart, characterized by resistance to conventional chemotherapy and poor prognosis of the patients [2]. Therefore, biomarkers related to tumor progression and chemoresistance remain to be investigated and may become potential targets for future therapy in T-cell leukemia/lymphoma.

MicroRNAs (miRs), a class of 19- to 23-nucleotide noncoding RNA molecules, regulate gene expression by targeting mRNA at the 3′ untranslated region (UTR) [3]. Growing evidences suggested that miRs are critical regulators in tumorigenesis and drug resistance [4, 5]. MiR181 is essential for lymphocyte differentiation and maturation in thymus [6]. More recently, it has been reported that miR181 overexpression promotes cell proliferation and activates PI3K/AKT signaling transduction pathway [7, 8]. Activated in lymphoid malignancies [9], AKT plays a pivotal role in tumor progression and resistance to chemotherapeutic agents [10, 11]. Here we assessed miR181a expression, as well as its relation to AKT activation and chemoresistance in T-cell leukemia/lymphoma.

## 2. Patients and Methods

### 2.1. Patients

One hundred and thirty patients diagnosed with T-ALL or T-cell lymphoma were enrolled in this study, including 32 T-ALL, 16 T-LBL, 45 PTCL-NOS, 15 ALCL, and 22 AITL. Histologic diagnoses were established according to the WHO classification [1]. PTCL cases (PTCL-NOS, ALCL, and AITL) were treated with CHOP-based chemotherapy. T-LBL and T-ALL cases were treated with HyperCVAD-A/B regimens as previously reported [12, 13]. Response rates were assessed according to the criteria as reported [12, 13]. The clinicopathological data of the patients was listed in Table 1. Thirty-four age- and sex-matched cases with reactive hyperplasia were referred to as controls. The study was approved by the Institutional Review Board with informed consent obtained in accordance with the Declaration of Helsinki.

### 2.2. Cell Lines and Reagents

T-leukemia/lymphoma cell lines Jurkat, H9, and embryonic kidney cell line HEK-293T were available from American Type Culture Collection (Manassas, VA, USA). Doxorubicin-resistant Jurkat and H9 cells were established by exposure to gradually increasing concentrations of doxorubicin* in vitro*, as described by Huang et al. [14].

### 2.3. Cell Proliferation Assay

Cell proliferation was measured by MTT and EdU incorporation assay. Cells were seeded in 96-well plates and incubated with the indicated concentrations of reagents at 37°C. After 72 h incubation, 0.1 mg of MTT was added to each well and the absorbance was measured at 490 nm by spectrophotometry. EdU assay was conducted using Cell-Light EdU imaging kit (RiboBio, Guangzhou, China) according to the manufacturer's instruction.

### 2.4. MiR181a Detection

Total RNA was extracted from 20 *μ*m thick paraffin (*n* = 100) or frozen sections (*n* = 64) using RecoverAll total nucleic acid isolation kit or Trizol agent following the manufacturer's protocol. MiR181a expression was analyzed by real-time quantitative RT-PCR using miRNA reverse transcription kit, hsa-miR 181a assay, and 7500HT Fast Real-time PCR system (Applied Biosystems, CA, USA). RNU24 was used as endogenous control and Jurkat cells for calibration. A relative quantification was calculated using the ^ΔΔ^CT method [15].

### 2.5. Western Blot

Cells were lysed in 200 *μ*L lysis buffer (0.5 M Tris-HCl, pH 6.8, 2 mM EDTA, 10% glycerol, 2% SDS, and 5% *β*-mercaptoethanol). Protein extracts (20 *μ*g) were electrophoresed on 10% SDS polyacrylamide gels and transferred to nitrocellulose membranes. Membranes were blocked with 5% nonfat dried milk in Tris-buffered saline and incubated for 2 h at room temperature with appropriate primary antibody, followed by horseradish peroxidase-conjugated secondary antibody. The immunocomplexes were visualized using chemiluminescence phototope-horseradish-peroxidase kit. Actin was used to ensure equivalent protein loading. Antibodies against phosphorylated-AKT (p-AKT), AKT, actin, and chemiluminescence phototope-horseradish-peroxidase kit were obtained from Cell Signaling (Beverly, MA, USA). Anti-PTEN antibody was from Abcam (Cambridge, UK). Horseradish peroxidase-conjugated goat anti-mouse-IgG and goat anti-rabbit-IgG antibodies were from Santa Cruz Biotechnology (Santa Cruz, CA, USA).

### 2.6. Cell Transfection

HEK-293T cells were incubated with pEZX-181a vector (HmiR0292-MR03) or a control vector pEZX-ct (CmiR0001-MR03, Genecopia, MD, USA) and Lipofectamine 2000 (Invitrogen, CA, USA) for 24 h and replaced in fresh medium for further experiments. To inhibit miR181a expression, Jurkat and H9 cells were transfected with 10 nM antagomir using Lipofectamine 2000 (Invitrogen) for 24 h. The miR181a antagomir and the negative control were synthesized by Shanghai Biotend Biotechnologies Co., Ltd. (Shanghai, China).

### 2.7. Immunohistochemistry Assay

Immunohistochemistry was performed on 5 *μ*m paraffin sections with an indirect immunoperoxidase method using antibodies against p-AKT (Cell Signaling). Expression levels were scored semiquantitatively based on percentage of positive cells: +, <25%; ++, 25–49%; +++, 50–74%; ++++, 75–100%.

### 2.8. Statistical Analysis

Differences of miR181a expression among groups were assessed using Mann-Whitney *U* test. The association between miR181a and p-AKT expression in human tumor samples was analyzed by Fisher's exact test.* In vitro* experimental results were expressed as mean ± S.D. of data obtained from three separate experiments and determined using *t*-test to compare variance. *P* < 0.05 was considered statistically significant.

## 3. Results

### 3.1. MiR181a Was Overexpressed in T-Cell Leukemia/Lymphoma and Related to AKT Activation

**Figure 1:**
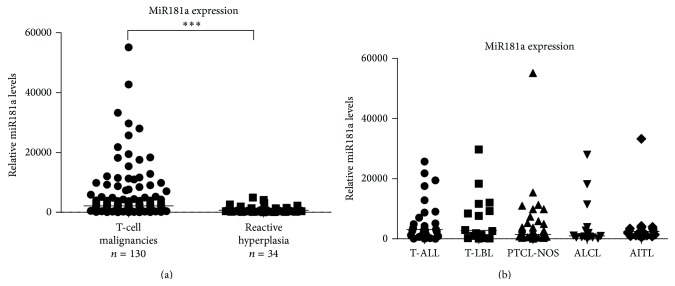
MiR181a was overexpressed in T-cell leukemia/lymphoma. (a) As detected by real-time quantitative PCR, miR181a was overexpressed in T-cell malignancies. ^***^ *P* < 0.001 comparing with reactive hyperplasia. The relative expression level of each patient was calculated based on the lowest expression value. (b) MiR181a was overexpressed in acute T-cell lymphoblastic leukemia (T-ALL, *n* = 32), as well as subtypes of T-cell lymphoma, including T-cell lymphoblastic lymphoma (T-LBL, *n* = 16), peripheral T-cell lymphoma, not otherwise specified (PTCL-NOS, *n* = 45), anaplastic large cell lymphoma (ALCL, *n* = 15), and angioimmunoblastic T-cell lymphoma (AITL, *n* = 22).

Compared with reactive hyperplasia, miR181a was overexpressed in T-cell leukemia/lymphoma (*P* < 0.0001, Figure 1(a)). No significant difference was observed among T-ALL and subtypes of T-cell lymphoma (*P* = 0.5153, Figure 1(b)).

**Figure 2:**
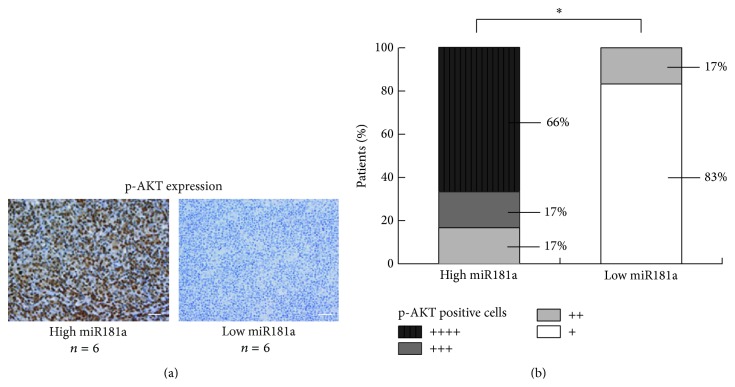
MiR181a overexpression was related to AKT activation in T-cell leukemia/lymphoma. As revealed by immunohistochemistry (a), increased positivity of p-AKT was observed in primary tumor samples of T-cell lymphoma patients with high miR181a expression (*n* = 6), compared to those with low miR181a expression (*n* = 6) (b). ^*^ *P* < 0.05 comparing with low miR181a expression. Bar = 50 *μ*m.

The median value of relative miR181a expression in T-cell leukemia/lymphoma was 2136. The patients with miR181a expression level over and equal to the median value were regarded as high miR181a expression, whereas those below the median value were included in the low miR181a expression. Patients with high miR181a expression had significantly lower overall response rate (ORR) than those with low miR181a expression (Table 1). P-AKT expression was detected by immunohistochemistry in primary tumor sections of 12 T-cell lymphoma patients (6 cases from high miR181a expression group and 6 cases from low miR181a expression group, Figure 2(a)). High miR181a expression was associated with increased positivity of p-AKT (*P* = 0.0152, Figure 2(b)).

### 3.2. MiR181a Promoted Cell Proliferation and Induced Chemoresistance through Activating AKT

**Figure 3:**
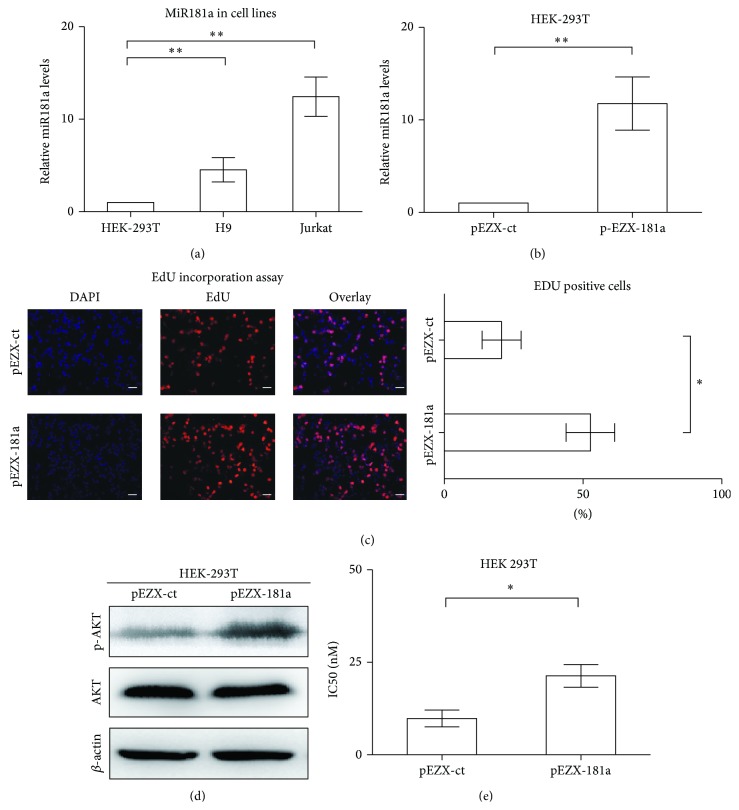
Ectopic expression of miR181a enhanced cell proliferation and resistance to doxorubicin through AKT activation. (a) T-leukemia/lymphoma Jurkat and H9 cells had significantly higher expression levels of miR181a than that of HEK-293T cells. ^**^ *P* < 0.01 comparing with HEK-293T cells. (b) Transfection with miR181a (pEZX-181a) in HEK-293T cells resulted in significantly increased miR181a expression. ^**^ *P* < 0.01 comparing with the control pEZX-ct cells. (c) EdU incorporation assay in HEK-293T cells showed that miR181a-overexpressing pEZX-181a cells presented with increased EdU-positive cells. ^*^ *P* < 0.05, comparing with the control pEZX-ct cells. Bar = 20 *μ*m. (d) Overexpression of miR181a increased AKT phosphorylation, while the total protein level remained constant. (e) IC50 of doxorubicin was significantly higher in the pEZX-181a cells than in the control pEZX-ct cells. ^*^ *P* < 0.05 comparing with the control pEZX-ct cells.

T-leukemia/lymphoma cell lines Jurkat and H9 possessed higher levels of miR181a expression than that of HEK-293T cells (*P* = 0.0023 and *P* = 0.0030, resp., Figure 3(a)). To gain insight into the biological function of miR181a, HEK-293T cells, with lowest miR181a expression, were transiently transfected with miR181a (pEZX-181a, Figure 3(b)). Ectopic expression of miR181a remarkably accelerated cell growth, as compared to the control cells (pEZX-ct). In parallel with increased cell proliferation, the percentage of EdU-positive cells was significantly higher in pEZX-181a cells (52.7% ± 8.7%) than in pEZX-ct cells (20.7% ± 7.0%, *P* = 0.0458, Figure 3(c)). Of note, overexpression of miR181a increased AKT phosphorylation, while the total protein level remained constant (Figure 3(d)). AKT is the key regulator of cell proliferation and drug resistance [11, 16]. Accordingly, the IC50 of doxorubicin was significantly increased in the miR181a-overexpressing HEK-293T cells, as compared to the control cells (21.3 ± 3.1 nM versus 9.8 ± 2.3 nM, *P* = 0.0260, Figure 3(e)).

### 3.3. MiR181a Overexpression Corresponded to Chemoresistance in T-Leukemia/Lymphoma Cells

**Figure 4:**
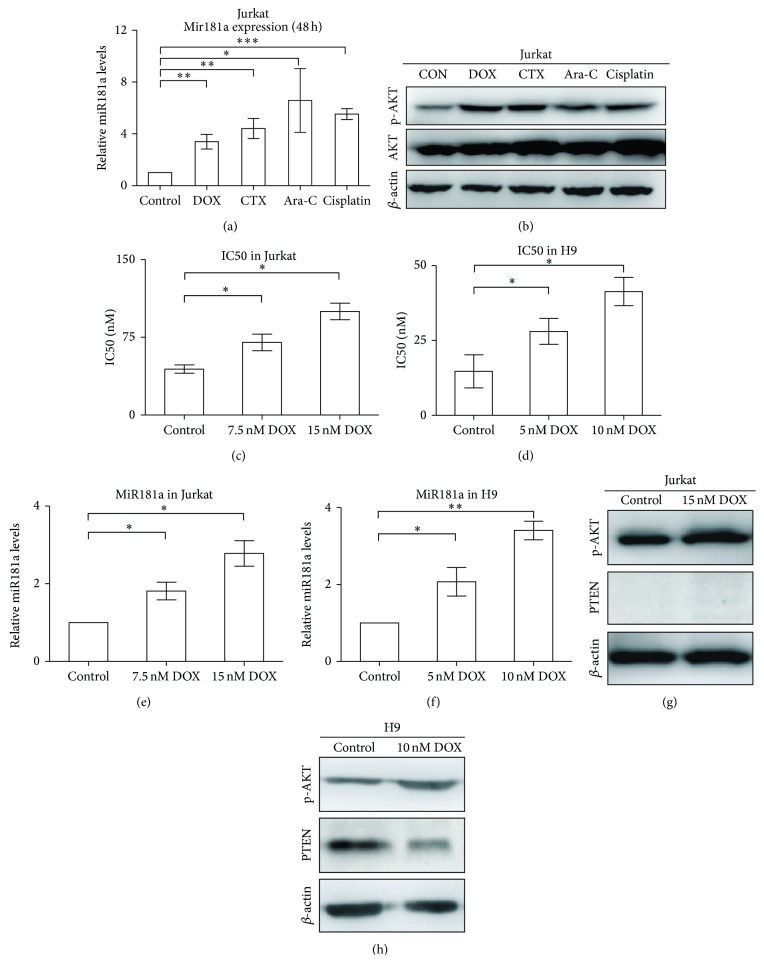
Exposure of T-leukemia/lymphoma cells to chemotherapeutic agents upregulated miR181a expression and resulted in AKT activation. (a) When Jurkat cells were treated with chemotherapeutic agents for 48 h, miR181a expression was significantly increased. CON, untreated; DOX, doxorubicin; CTX, cyclophosphamide; Ara-C, cytarabine. ^*^ *P* < 0.05; ^**^ *P* < 0.01; ^***^ *P* < 0.001 comparing with the CON cells. (b) In accordance with miR181a upregulation, increased AKT phosphorylation was observed by western blot, while the total protein level remained constant. (c) and (d) IC50 of doxorubicin was significantly increased in doxorubicin-resistant Jurkat (c) and H9 (d) cells, which were exposed to doxorubicin for 3 weeks. (e) and (f) MiR181a expression was significantly increased in doxorubicin-resistant Jurkat (e) and H9 (f) cells. CON, untreated; ^*^ *P* ≤ 0.05; ^**^ *P* ≤ 0.01 comparing with the CON cells. (g) and (h) P-AKT expression was significantly increased in doxorubicin-resistant Jurkat (g) and H9 (h) cells.

Doxorubicin (DOX), cyclophosphamide (CTX), cytarabine (Ara-C), and cisplatin are main chemotherapeutic agents used in treating T-cell malignancies. When Jurkat cells were exposed to these agents for 48 h, miR181a expression was significantly increased (*P* = 0.0019, *P* = 0.0016, *P* = 0.0172, and *P* < 0.0001, resp., Figure 4(a)), in accordance with increased AKT phosphorylation detected by Western blot (Figure 4(b)).

Acquired drug resistance is an important obstacle that impairs the success of cancer treatment. Isogenic doxorubicin-resistant sublines were developed as previously reported [14, 17] at the concentrations of 7.5 nM (Jurkat/7.5 nM DOX) and 15 nM (Jurkat/15 nM DOX) in Jurkat cells, as well as 5 nM (H9/5 nM DOX) and 10 nM (H9/10 nM DOX) in H9 cells. Compared with the parental cells (Jurkat cells, 44.3 ± 4.0 nM; H9 cells, 14.7 ± 5.5 nM), IC50 of doxorubicin was significantly increased in Jurkat/7.5 nM DOX and Jurkat/15 nM DOX cells (70.1 ± 8.0 nM and 100.0 ± 8.0 nM, *P* = 0.0453 and *P* = 0.0152, resp., Figure 4(c)) and in H9/5 nM DOX and H9/10 nM DOX cells (28.0 ± 4.3 nM and 41.3 ± 4.7 nM, *P* = 0.0481 and *P* = 0.0443, resp., Figure 4(d)). Accordingly, levels of miR181a expression were significantly higher in doxorubicin-resistant cells than in the parental cells (1.8 ± 0.2-fold in Jurkat/7.5 nM DOX and 2.8 ± 0.3-fold in Jurkat/15 nM DOX cells, *P* = 0.0253 and *P* = 0.0112, Figure 4(e); 2.1 ± 0.6-fold in H9/5 nM and 3.4 ± 0.2-fold in H9/10 nM DOX cells, *P* = 0.0384 and *P* = 0.0032, resp., Figure 4(f)), consistent with AKT activation (Figures 4(g) and 4(h)). Increased miR181a expression was linked to AKT phosphorylation, not only in PTEN-negative Jurkat cells [18] but also in PTEN-positive H9 cells (Figures 4(g) and 4(h)). Therefore, regulation of AKT phosphorylation could be independent of PTEN expression in T-cell leukemia/lymphoma. Further drug-sensitivity test showed that these resistant sublines with miR181a overexpression also had cross-resistance to other chemotherapeutic agents (Table 2). Therefore, exposure to chemotherapeutic agents could induce miR181a expression and AKT activation, which is closely related to acquired chemoresistance.

### 3.4. MiR181a Inhibition Enhanced T-Leukemia/Lymphoma Cell Sensitivity to Chemotherapeutic Agents

**Figure 5:**
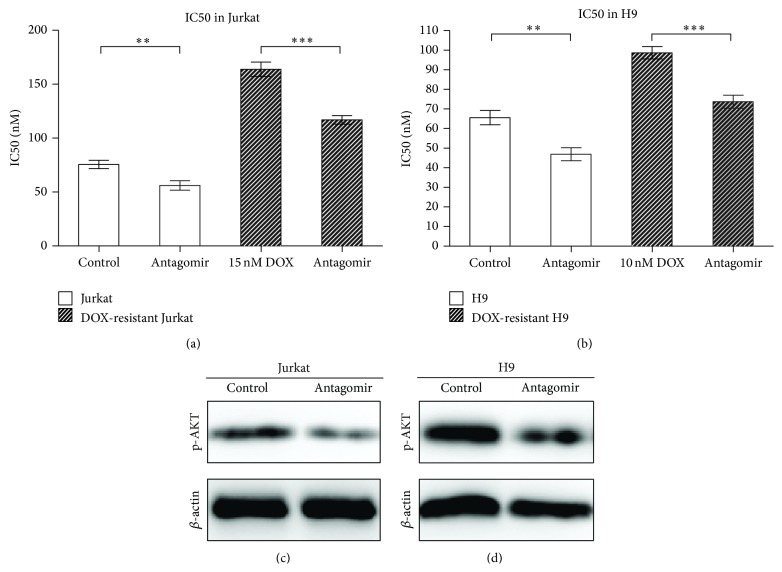
Specific inhibition of miR181a expression could increase T-leukemia/lymphoma cells and resistant sublines sensitivity to doxorubicin, along with p-AKT downregulation. (a) and (b) IC50 of doxorubicin was significantly decreased in antagomir-treated Jurkat (a) and H9 (b) cells. The DOX-resistant Jurkat and H9 cells were exposed to doxorubicin for 6 weeks. ^**^ *P* ≤ 0.01; ^***^ *P* ≤ 0.001 comparing with the control cells. (c) and (d) Decreased AKT phosphorylation was found in miR181a antagomir-treated Jurkat (c) and H9 (d) cells.

Specific inhibition of miR181a in Jurkat and H9 cells as well as their resistant sublines, using an antagomir, significantly increased cell sensitivity to doxorubicin (Figures 5(a) and 5(b)) and decreased AKT phosphorylation (Figures 5(c) and 5(d)). Similar to doxorubicin, decreased miR181a expression was also related to reduced IC50 of cisplatin and cyclophosphamide in doxorubicin-resistant Jurkat and H9 cells (Table 3).

## 4. Discussion

In addition to genetic abnormalities, epigenetic aberrations, particularly those of miRs, participate in human carcinogenesis [5, 19]. MiR181a is critically involved in hematological malignancies. In acute myelogenous leukemia, increased miR181a expression was significantly associated with a higher complete remission rate of the patients [20]. However, as recently reported in lymphoid malignancies like acute lymphoblastic leukemia and multiple myeloma, miR181 overexpression was related to advanced stage and tumor progression [7, 21]. In accordance with malignant T cells, miR181a is upregulated in normal T-cell counterpart and miR181a/b-deficient mice show severe defects in T-cell development [6, 8]. Therefore, miR181 may have different roles in hematological malignancies. Our study showed that miR181a, independent of histological subtypes, was overexpressed and these patients were less responding to treatment, referring to miR181a as a common biomarker of chemoresistance in T-cell leukemia/lymphoma.

Chemoresistance, determining therapeutic effect and clinical outcome of the patients, is one of the control factors in cancer treatment, including T-cell leukemia/lymphoma [22]. Here miR181a was closely related to chemoresistance in T-leukemia/lymphoma. This was consistent with previous reports in B-cell lymphoma that high expression of miR181 could lead to decreasing proapoptotic protein Bim and increasing resistance to chemotherapy [23]. AKT is a key tuning point in tumor cell growth and chemosensitivity [24, 25]. MiR181 is a central regulator of PI3K pathway, since miR181a/b-deficient mice showed severe defects in lymphoid development and T-cell homeostasis associated with impaired PI3K/AKT cascade [8]. As mechanism of action, miR181 targets PTPN, DUSP5, and DUSP6, resulting in PI3K/AKT activation and tumorigenesis in murine T-cell leukemia [7, 8]. Our results showed that ectopic expression of miR181a leads to AKT phosphorylation, enhancing cell proliferation and inducing cell resistance to chemotherapy in T-cell leukemia/lymphoma. This correlation of miR181a overexpression with AKT activation was observed not only in cell lines but also in primary tumor samples of patients with T-leukemia/lymphoma. Apart from primary chemoresistance, acquired chemoresistance is also an important factor of treatment failure. MiR181a expression of Jurkat cells was significantly upregulated after exposure to chemotherapeutic agents and linked to increased AKT phosphorylation. Meanwhile, isogenic doxorubicin-resistant cell lines were developed, which were resistant to doxorubicin and had cross-resistance to other chemotherapeutic drugs. The relative resistance to chemotherapeutic agents was along with increased miR181a expression and subsequent AKT activation, further confirming that miR181a induced AKT activation and contributed to chemoresistance in T-cell leukemia/lymphoma.

## 5. Conclusions

MiR181a was involved in T-cell leukemia/lymphoma through modulation of AKT pathway. Functioned as a critical regulator of chemosensitivity, miR181a could thus be a promising therapeutic target in treating T-cell malignancies resistant to chemotherapy.

## Figures and Tables

**Table 1:** Clinicopathological characteristics of 130 patients with T-cell leukemia/lymphoma.

Characteristics	High miR181a expression (*n* = 65)	Low miR181a expression (*n* = 65)	*P* value
Age (years)			
≥60	13	17	0.5328
<60	52	48	
Gender			
Male	42	32	0.1106
Female	23	33	
Lactic dehydrogenase level (LDH)			
Normal	19	23	0.5740
Above normal	46	42	
Pathological subtypes			
T-ALL	15	17	0.6656
T-LBL	8	8	
PTCL-NOS	22	23	
ALCL	6	9	
AITL	14	8	
International prognostic index (IPI)			
Low	12	11	0.3152
Low/intermediate	23	33	
Intermediate/high	20	15	
High	10	6	
Overall response (CR + PR)	38	50	0.0385

^*^CR, complete remission; PR, partial remission.

**Table 2:** Cross-resistance of doxorubicin-resistant cells to other chemotherapeutic agents.

Agents	IC50		*P* value	IC50		*P* value
	Jurkat	Doxorubicin-resistant Jurkat		H9	Doxorubicin-resistant H9	
Cisplatin (uM)	6.2 ± 0.3	9.0 ± 0.4	0.0039	5.7 ± 0.3	8.5 ± 0.5	0.0067
Cyclophosphamide (mM)	3.3 ± 0.2	4.8 ± 0.2	0.0056	2.4 ± 0.2	3.9 ± 0.3	0.0106

**Table 3:** Inhibition miR181a expression sensitized T-leukemia/lymphoma cells to chemotherapeutic agents.

Agents	IC50		*P* value	IC50		*P* value	IC50		*P* value	IC50		*P* value
	Jurkat control	Jurkat antagomir		Doxorubicin-resistant Jurkat control	Doxorubicin-resistant Jurkat antagomir		H9 control	H9 antagomir		Doxorubicin-resistant H9 control	Doxorubicin-resistant H9 antagomir	
Cisplatin (uM)	6.6 ± 0.2	5.1 ± 0.1	0.0014	9.0 ± 0.4	7.0 ± 0.4	0.0215	5.7 ± 0.3	4.4 ± 0.2	0.0264	8.5 ± 0.5	6.0 ± 0.5	0.0191
Cyclophosphamide (mM)	3.3 ± 0.2	1.6 ± 0.2	0.0018	4.8 ± 0.2	3.6 ± 0.2	0.0214	2.4 ± 0.2	0.9 ± 0.1	0.0019	3.9 ± 0.3	2.5 ± 0.2	0.0134
